# Reduced Skeletal Muscle Mass Is Associated with an Increased Risk of Asthma Control and Exacerbation

**DOI:** 10.3390/jcm11237241

**Published:** 2022-12-06

**Authors:** Shuwen Zhang, Xin Zhang, Ke Deng, Changyong Wang, Lisa G. Wood, Huajing Wan, Lei Liu, Ji Wang, Li Zhang, Ying Liu, Gaiping Cheng, Peter G. Gibson, Brian G. Oliver, Fengming Luo, Vanessa M. McDonald, Weimin Li, Gang Wang

**Affiliations:** 1 Pneumology Group, Department of Integrated Traditional Chinese and Western Medicine, West China Hospital, Sichuan University, Chengdu 610041, China; 2 Department of Respiratory and Critical Care Medicine, Clinical Research Center for Respiratory Disease, West China Hospital, Sichuan University, Chengdu 610041, China; 3 Laboratory of Pulmonary Immunology and Inflammation, Frontiers Science Center for Disease-Related Molecular Network, Sichuan University, Chengdu 610041, China; 4 Priority Research Center for Healthy Lungs, Hunter Medical Research Institute, The University of Newcastle, New Lambton, NSW 2308, Australia; 5 Department of Clinical Nutrition, West China Hospital, Sichuan University, Chengdu 610041, China; 6 Centre of Excellence in Severe Asthma and Priority Research Centre for Healthy Lungs, Hunter Medical Research Institute, The University of Newcastle, Newcastle, NSW 2300, Australia; 7 School of Life Sciences, University of Technology Sydney, Ultimo, NSW 2007, Australia; 8 Woolcock Institute of Medical Research, The University of Sydney, Sydney, NSW 2000, Australia; 9 Respiratory Microbiome Laboratory, Frontiers Science Center for Disease-Related Molecular Network, Sichuan University, Chengdu 610041, China

**Keywords:** asthma, skeletal muscle mass, exacerbation, clinical prediction model

## Abstract

Background: Skeletal muscle mass (SMM) has been suggested to be associated with multiple health-related outcomes. However, the potential influence of SMM on asthma has not been largely explored. Objective: To study the association between SMM and clinical features of asthma, including asthma control and exacerbation, and to construct a model based on SMM to predict the risk of asthma exacerbation (AEx). Methods: In this prospective cohort study, we consecutively recruited patients with asthma (*n* = 334), classified as the SMM ^Normal^ group (*n* = 223), SMM ^Low^ group (*n* = 88), and SMM ^High^ group (*n* = 23). We investigated the association between SMM and clinical asthma characteristics and explored the association between SMM and asthma control and AEx within a 12-month follow-up period. Based on SMM, an exacerbation prediction model was developed, and the overall performance was externally validated in an independent cohort (*n* = 157). Results: Compared with the SMM ^Normal^ group, SMM ^Low^ group exhibited more airway obstruction and worse asthma control, while SMM ^High^ group had a reduced eosinophil percentage in induced sputum. Furthermore, SMM ^Low^ group was at a significantly increased risk of moderate-to-severe exacerbation compared with the SMM ^Normal^ group (relative risk _adjusted_ 2.02 [95% confidence interval (CI), 1.35–2.68]; *p* = 0.002). In addition, a model involving SMM was developed which predicted AEx (area under the curve: 0.750, 95% CI: 0.691–0.810). Conclusions: Low SMM was an independent risk factor for future AEx. Furthermore, a model involving SMM for predicting the risk of AEx in patients with asthma indicated that assessment of SMM has potential clinical implications for asthma management.

## 1. Introduction

Asthma is a common chronic respiratory disease affecting 1–18% of the population in different countries [1] Uncontrolled asthma may lead to reduced physical activity in daily life [2]. Lack of physical activity and other risk factors such as aging, nutritional status, and chronic inflammation could contribute to progressive loss of skeletal muscle mass (SMM) [3]. Reduced SMM is related to functional comorbidities, including mobility disorders, risk of falls and fractures, and loss of physical independence in activities of daily living for patients with asthma, which would increase the demand on the healthcare system [3]. Bioelectrical impedance analysis (BIA), as an inexpensive and non-invasive technique, provides measurements of SMM with little complexity [4].

SMM has been shown to affect health outcomes differentially. A decrease in muscle mass has been linked to greater insulin resistance and protection against the development of type 2 diabetes [5]. In contrast, lower amounts of SMM are associated with numerous health problems. A recently published study suggested that a lower muscle mass leads to an increased risk of cardiovascular events [6]. Moreover, the substantial loss of muscle mass relative to fat mass, termed “sarcopenia”, has been found to have a negative effect on the quality of life and survival of patients [7,8,9]. This relationship has been observed not only in the elderly population and in cancer patients, in whom muscle loss is prevalent, but also in the general population [3,10].

There is increasing evidence that the loss of SMM is associated with lung health [11,12]. This may be relevant to asthma and chronic obstructive pulmonary disease (COPD), both of which are chronic inflammatory airway diseases [13]. Reduced SMM has been associated with impaired lung function and poor health status in patients with COPD [14,15,16]. We and others have shown that various extra-pulmonary traits, including obesity, are associated with asthma control [17,18,19]. However, to date, no studies have specifically examined SMM as a potential extra-pulmonary treatable trait in asthma outcome in future.

In this prospective cohort study, we explored whether reduced SMM was associated with worse asthma control and exacerbation (AEx). Subsequently, a prediction model involving SMM for identifying the risk of AEx was developed. The relative importance of SMM for all screened predictors in the model for predicting AEx was also assessed.

## 2. Materials and Methods

### 2.1. Study Design and Patients

The ASAN (https://www.severeasthma.org.au, accessed on 3 December 2022) is a multicenter clinical research network (Australia, Singapore, China, and New Zealand) in a real-world setting. This prospective cohort study consecutively recruited adult patients (aged ≥ 18 years) diagnosed with stable asthma at the West China Hospital, Sichuan University. Asthma was diagnosed based on the Global Initiative for Asthma [1]. Stable asthma was defined as no respiratory tract infections, asthma exacerbations, or systemic corticosteroid (SCS) use in the previous 4 weeks. Patients who were unable to complete the questionnaires, perform spirometry, perform the sputum induction, or were pregnant or breastfeeding were excluded from the study.

We recruited 334 patients (recruitment period: March 2014 to October 2018). According to the age, sex, height, and weight of the individual, the range of normal values for SMM in Asian populations was calculated using a validated equation by multifrequency BIA and classified as SMM ^Normal^ group [20,21]. SMM values lower than the 10th percentile of the reference values were classified as SMM ^Low^ group and values of SMM equal to or higher than the 90th percentile of the reference values were classified as SMM ^High^ group [20,21]. These patients were followed for 12 months to assess the occurrence of AEx. We used these participants as the training cohort to explore the association between SMM and asthma and construct a prediction model.

**Figure 1:**
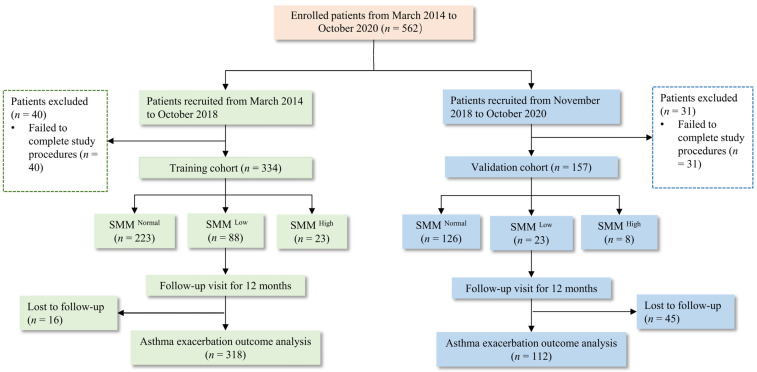
Flowchart for patient inclusion in the training and validation cohorts. SMM, skeletal muscle mass.

We independently recruited 157 patients as the validation cohort (recruitment period: November 2018 to October 2020) to validate the prediction model established in the training cohort (Figure 1). As in the training cohort, these patients were also followed up for 12 months to assess the occurrence of AEx. All patients (training and validation cohorts) were included in this study satisfying the above inclusion/exclusion criteria.

All patients provided written informed consent before participating in the study. The study was approved by the Institutional Review Board of West China Hospital, Sichuan University (Chengdu, China) (2014-30) and registered in the Chinese Clinical Trial Registry (ChiCTR-OOC-16009529; http://www.chictr.org.cn, accessed on 3 December 2022).

### 2.2. Data Collection and Clinical Assessments

Participants were followed up for 1 year with visits at baseline, 1 month, 3 months, 6 months, 9 months and 12 months.

Baseline data of the participants were collected, which included demographics, medications at/prior to study entry, asthma history, atopy, BMI, asthma control questionnaire-6 (ACQ-6), psychological status assessed using the Hospital Anxiety and Depression Scale (HADS) [22]. And all included subjects underwent spirometry, sputum induction, fractional exhaled nitric oxide (FeNO), blood sampling and body composition (BC).

### 2.3. Anthropometric and BCAssessments

SMM was measured as a parameter in BC assessments at baseline. BC was evaluated using BIA (InBody S10 analyzer; Biospace Co., Ltd., Seoul, Republic of Korea) according to the user manual [20]. Body resistance (R) was used to estimate the total body SMM according to the method previously described by Janssen et al. as follows [21]:

SMM (kg) = [(Ht^2^/R × 0.401) + (sex × 3.825) + (age × −0.071)] + 5.102

where Ht is height in centimeters and R is BIA resistance in ohms; for sex, men = 1 and women = 0, and age is in years.

Anthropometric and BC assessments were performed by trained nutritionists. The patients had overnight fasting, emptied their bladder by urinating, removed their clothes, and stood during the measurements, during which the ambient temperature remained at 25 degrees centigrade. Height and weight were measured to the nearest 0.1 cm and 0.1 kg when wearing light clothing and no shoes [23]. BMI was calculated (BMI = weight [kg]/height squared [m^2^]). Waist circumference and hip circumference were measured at the navel and maximum posterior protuberance of the buttocks, respectively.

BC variables, including visceral fat area (VFA) (cm^2^), fat mass (FM) (kg), percentage body fat (PBF), and SMM (kg), were estimated.

### 2.4. Spirometry and FeNO

Spirometry was performed at baseline according to the American Thoracic Society/European Respiratory Society (ATS/ERS) standards using a spirometer (Med Graphics CPES/D USB, St. Paul, MN, USA) [24]. Pre-bronchodilator FEV_1_ and pre-bronchodilator forced vital capacity (FVC) were also measured. The largest pre-bronchodilator FEV_1_ and FVC values from the three forced expiratory curves were used for analysis [24]. We measured FeNO before spirometry testing using a NIOX analyzer (Aerocrine, Solna, Sweden) in accordance with the ATS/ERS recommendations [25].

### 2.5. Sputum and Blood Processing

Sputum induction, processing, and blood analyses were performed at baseline as described in our previous studies [26,27]. The total and differential blood cell counts and serum immunoglobulin E (IgE) levels were measured. Details are provided in the Supplementary Materials.

### 2.6. Atopy

Atopy was confirmed at baseline by at least one positive skin prick test (SPT) of common allergens, defined as a wheal diameter ≥ 3 mm after 15 min. The details are provided in the Supplementary Materials [28].

### 2.7. Asthma Control

Asthma control was assessed using the ACQ-6 at baseline [29]. The ACQ score is the mean of the six items and ranges from zero (totally controlled) to six (severely uncontrolled). A mean score of ≥ 0.75 is indicative of partially controlled or uncontrolled asthma. In our study, the patients were dichotomized into 2 groups on the basis of ACQ scores. We labeled those with scores of less than 0.75 as the well-controlled asthma and those with scores of more than 0.75 as the incompletely controlled asthma.

### 2.8. Asthma Exacerbation

We have collected the exacerbation history of all patients at baseline and follow-up for 1 year at 3 months, 6 months, 9 months and 12 months to assess exacerbations (face-to-face visits or telephone calls if unable to attend). An asthma exacerbation was defined based on ATS/ERS statement [30].

The definition of a severe asthma exacerbation for clinical trials should include at least one of the following: (a) use of SCS (tablets, suspension, or injection), or an increase from a stable maintenance dose, for at least 3 days. For consistency, courses of corticosteroids separated by 1 week or more were treated as separate severe exacerbations and (b) a hospitalization or emergency room (ER) visit because of asthma, requiring SCS [30].

The definition of a moderate asthma exacerbation included one or more of the following: deterioration in symptoms, deterioration in lung function, and increased rescue bronchodilator use. These features lasted for 2 days or more, but not be severe enough to warrant SCS use and/or hospitalization. ER visits for asthma (e.g., for routine sick care) that do not require SCS were classified as moderate exacerbations [30].

### 2.9. Statistical Analyses

For categorical data, descriptive variables were presented as *n* (%). Continuous data were presented as means with standard deviations or medians with interquartile ranges, depending on the distribution assessed by the Kolmogorov–Smirnov test. Differences in continuous data between the training and validation cohorts were assessed using the Mann–Whitney *U* test. The differences between the three groups were evaluated using one-way analysis of variance or Kruskal–Wallis *H* test for continuous variables, and the Chi-square test or Fisher’s exact test for categorical variables, as appropriate.

In addition, *post-hoc* Bonferroni comparisons were performed, with the cutoff for significance set at α/*n* (α = 0.05, where *n* is the number of comparisons). Logistic regression was used to assess the associations between SMM and asthma control at baseline (odds ratio [OR], 95% confidence interval [95% CI]), SMM and AEx during follow-up (relative risk [RR], 95% CI). Considering that COPD, obstructive sleep apnea, bronchiectasis, diabetes, obesity, and gastroesophageal reflux disease (GERD) may be confounding factors in the relationship between SMM and AEx [31,32,33], multivariable logistic regression by backward elimination in a stepwise fashion and sensitivity analysis was used to explore the effect of confounders on the results of the analysis. The above analyses were conducted using SPSS version 26.0 (IBM Corp., Armonk, NY, USA). In all statistical analyses, a *p* value of less than 0.05 was considered statistically significant.

### 2.10. Clinical Prediction Model for Predicting AEx

#### 2.10.1. Selection of Variables and Clinical Prediction Model Establishment

We used a logistic model to select the variables to construct a prediction model [34]. We then calculated the area under the receiver operator characteristic (ROC) curve (AUC) to determine how many candidate factors should be chosen [35]. The details are provided in the Supplementary Materials [36].

Multivariable logistic regression was incorporated in the training cohort, combining significant predictors from the least absolute shrinkage and selection operator (LASSO) method into one final model [37]. This model displayed RR and 95% CI. Each predictor’s contribution in the full model was measured as the partial chi-square statistic minus the predictor degrees of freedom [38].

#### 2.10.2. Nomogram Establishment of Predicting AEx

Relationships among predictors in the model were visualized using a nomogram [39], which maps the predicted probabilities into points on a scale from 0 to 100 in a user-friendly graphical interface [40]. In this study, we established a nomogram for predicting AEx, named “AEx nomogram”. The details are provided in the Supplementary Materials.

#### 2.10.3. Performance of the Model and Clinical Applicability of the Nomogram

The concordance index (C-index), Hosmer–Lemeshow (HL) goodness-of-fit test, and calibration curve were performed in the training and validation cohorts to estimate the prediction performance of the nomogram [41,42]. The internal and external validity of the model was determined. Internal validation was performed using 1000 bootstrap samplings to produce bias-corrected estimates of the model’s performance [43]. External validation was performed on a validation cohort. We then set the two models to further explore whether SMM improves the performance of the prediction model. Model 1 was adjusted for all the predictors from a prediction model minus SMM, while Model 2 was adjusted for all the predictors from a prediction model. The net reclassification improvement (NRI) (>0) can be viewed as an improvement in discrimination by adding SMM to the training and validation cohorts. Likewise, integrated discrimination improvement (IDI) (>0) was considered an improvement in discrimination by adding SMM in the two cohorts.

Finally, decision curve analysis (DCA) was conducted to determine the clinical usefulness of the AEx nomogram by quantifying the net benefits at different threshold probabilities in the training cohort [44,45]. To determine the applicability of SMM in the nomogram, we built three models: (A) a model containing only one variable-SMM, (B) an AEx nomogram with subtraction of SMM, and (C) an AEx nomogram. A clinical impact curve (CIC) was developed based on the DCA of a model (C) to visually display the estimated number of patients at a high risk of AEx for each risk threshold [46]. The details are provided in the Supplementary Materials.

Model performance, validation, and applicability were performed using R software (version 4.0.2; R Foundation for Statistical Computing, Vienna, Austria). The “glmnet” package was used for binary LASSO method, “rms” for nomogram and calibration curve plotting, “pROC” for AUC calculation, “PredictABEL” for NRI and IDI calculation, and “DecisionCurve” for decision curve analysis.

## 3. Results

### 3.1. Participant Characteristics

A total of 219 participants (65.6%) were female, with a median age of 44.0 (Q1, Q3: 35.0, 55.0) years and a median BMI of 23.02 (20.99, 25.02) kg/m^2^. The prevalence of family history of asthma and atopy was 38.5% and 35.9%, respectively. The median scores of HADS-D and HADS-A in these patients were 1.0 (0, 3.0) and 1.0 (0, 4.0), respectively; 6.6% (*n* = 22) participants had anxiety symptoms, and 7.5% (*n* = 25) had depression symptoms. There were 149 patients (44.6%) with incompletely controlled asthma and 96 patients (28.7%) had experienced at least one severe exacerbation in the last 12 months. Rhinitis (53.6%) and eczema (20.4%) were the most common comorbidities.

Of the 334 participants, 223 (66.8%) were classified into the SMM ^Normal^ group, 88 (26.3%) were classified into the SMM ^Low^ group, and 23 (6.9%) were classified into the SMM ^High^ group. Compared with the SMM ^Low^ group, SMM ^High^ group had less airway obstruction (1.64 [1.28, 2.49] vs. 2.32 [2.02, 2.65] L, *p* = 0.013 for pre-bronchodilator FEV_1_ in liters and 66.0 [47.0, 82.5] vs. 92.0 [78.0, 104.0] %, *p* < 0.001 for FEV_1_% predicted) (Table 1).

In addition, we explored the differences in inflammatory variables among the three groups. Participants with the SMM ^High^ group had fewer sputum eosinophils (0 [0, 0.25] vs. 0.25 [0, 3.50] %, *p* = 0.016) than those with the SMM ^Normal^ group. The peripheral blood cell counts showed no significant differences among the three groups (all *p* > 0.05). The IgE (*p* = 0.217) and FeNO (*p* = 0.464) levels did not differ significantly among the three groups (Table S1).

### 3.2. Anthropometric and BC Assessments

Compared with the SMM ^Normal^ group, SMM ^High^ group had a significantly higher waist-to-hip ratio (WHR) and BMI. The SMM ^Low^ group had a significantly lower WHR and BMI, while the SMM ^Low^ group had a significantly lower FM, PBF, and VFA than patients in the SMM ^Normal^ group (Table S2).

### 3.3. Asthma Control

There were significant differences in asthma control among the three groups. SMM ^High^ group had better asthma control than SMM ^Low^ group (ACQ-6 median scores, 0.2 [0, 0.6] vs. 0.8 [0.3, 1.3], *p* = 0.005). Logistic regression modeling showed that SMM ^Low^ group was at a significantly increased risk of incompletely controlled asthma (OR _adj_ 1.67 [95% CI: 1.01–2.75], *p* = 0.045) (Table 2).

### 3.4. Asthma Exacerbation

Compared with the SMM ^Normal^ group, SMM ^Low^ group had a greater proportion of participants experiencing severe AEx (13.6% vs. 24.4%, *p* = 0.006) and moderate-to-severe AEx (25.8% vs. 40.2%, *p* = 0.022) (Table 3).

**Figure 2:**
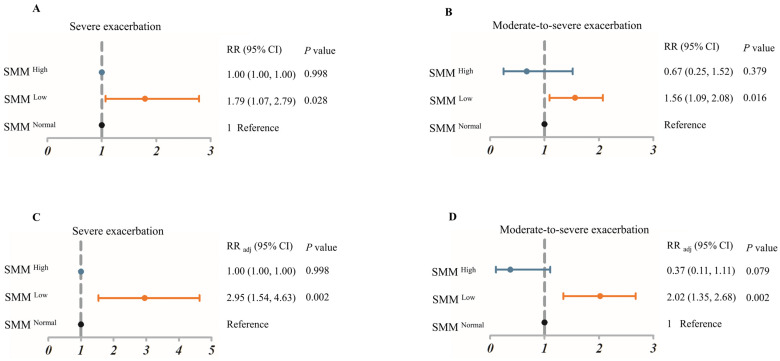
Associations of the SMM (skeletal muscle mass) with (**A**) severe exacerbation; univariate logistic regression analysis, (**B**) moderate-to-severe exacerbation, univariate logistic regression analysis. (**C**) severe exacerbation; multiple logistic regression analysis. Adjusted for age, sex, BMI, smoking status, ICS/LABA, cumulative doses of OCS equivalent to prednisone, severe exacerbation in the past year, pre-bronchodilator forced expiratory volume in 1 s% predicted (**D**) moderate-to-severe exacerbation, multiple logistic regression analysis, with normal SMM as the reference. Adjusted for age, sex, BMI, smoking status, ICS/LABA, cumulative doses of OCS equivalent to prednisone, severe exacerbation in the past year, pre-bronchodilator forced expiratory volume in 1 s% predicted. CI, confidence interval; RR, relative risk; RR _adj_, adjusted relative risk. Blue, SMM ^High^; Orange, SMM ^Low^; Black, SMM ^Normal^.

We further established logistic regression models to analyze the risk of AEx in the three groups. As a result, the SMM ^Low^ group had an increased risk of moderate-to-severe AEx adjusting for age, sex, BMI, smoking status, inhaled corticosteroids (ICS)/long-acting beta-agonist (LABA), cumulative doses of OCS equivalent to prednisone, pre-bronchodilator FEV_1_% predicted and moderate-to-severe asthma exacerbation last year (SMM ^Normal^ group as the reference; RR _adj_ 2.02 [95% CI: 1.35–2.68]; *p* = 0.002) (Figure 2).

Likewise, the SMM ^Low^ group was still significantly associated with moderate-to-severe AEx compared with the SMM ^Normal^ group adjusting confounders including age, sex, BMI, smoking status, ICS/LABA, cumulative doses of OCS equivalent to prednisone, pre-bronchodilator FEV_1_% predicted and severe asthma exacerbation last year, COPD, sleep apnea, bronchiectasis, diabetes, obesity, and GERD using multivariate logistic regression analysis with stepwise backward elimination (RR _adj_ 1.72 [95% CI: 1.19–2.29]; *p* = 0.006) (Table S3).

Additionally, after excluding the patients with COPD, sleep apnea, bronchiectasis, diabetes, obesity, and GERD, our sensitivity analysis indicated that this did not change the association between reduced SMM and AEx in the asthmatics (RR _adj_ 1.77 [95% CI: 1.06–2.53]; *p* = 0.032) (Table S4).

### 3.5. Clinical Prediction Model

#### 3.5.1. Selection of Variables and Establishment of a Clinical Prediction Model

**Figure 3:**
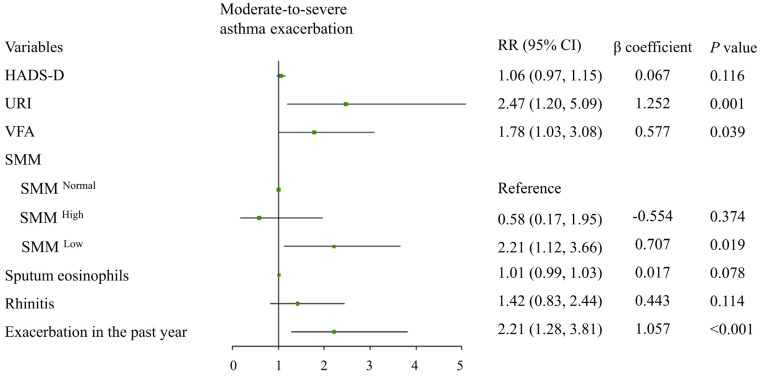
Risk factors in the prediction model for future moderate-to-severe exacerbation of asthma in the following years. Green dot, point estimate; Black line, 95% confidence interval.

In the training cohort, there were 17 variables with missing data (missing rates: 0.2% to 40.1%) (Table S5). The associations between 28 potential risk factors are shown in Table S6. Seven variables with nonzero coefficients in the LASSO method remained and were then included in the final multivariate logistic regression model (Figure S1), including low SMM (categorical variable) (RR 2.21 [95% CI: 1.12–3.66]; *p* = 0.019), VFA (categorical variable) (RR 1.78 [95% CI: 1.03–3.08]; *p* = 0.039), sputum eosinophils (continuous variable, %) (RR 1.01 [95% CI: 0.99–1.03]; *p* = 0.078), exacerbation in the past year (categorical variable) (RR 2.21 [95% CI: 1.28–3.81]; *p* < 0.001), rhinitis (categorical variable) (RR 1.42 [95% CI: 0.83–2.44]; *p* = 0.114), previous upper respiratory infection-induced asthma attack (categorical variable) (RR 2.47 [95% CI: 1.20–5.09]; *p* = 0.001), and HADS-D scores (continuous variable, score) (RR 1.06 [95% CI: 0.97–1.15]; *p* = 0.116) (Figure 3).

#### 3.5.2. Nomogram of Predicting AEx

**Figure 4:**
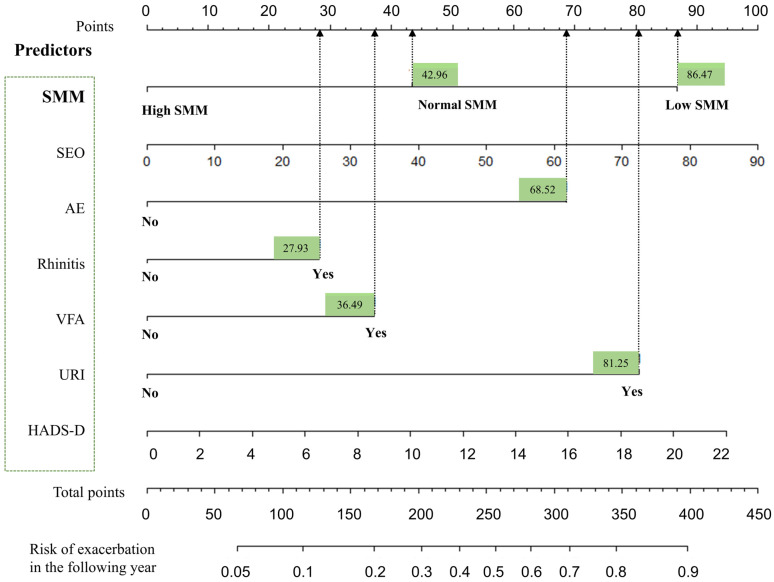
Asthma future exacerbation nomogram. The asthma future exacerbation nomogram was developed on the basis of established multivariable regression models in the whole cohort population. Using the nomogram, the probability of future asthma exacerbation in the following year can be estimated as follows. First, the judgment on predictor variables (e.g., yes or no, SMM ^Low^ or SMM ^High^) can be obtained from patients. Second, if a predictor is judged as “Yes”, the value of the predictor can be designated by drawing an upward straight line from “Yes” up to the “Points” line. Third, add up the points of all the predictors assessed as “Yes” to get the total points. Finally, the probability of future asthma exacerbation in the following year can be obtained by drawing a straight line from the “Total Points line” down to the “Risk of exacerbation in the following year” line. SMM, skeletal muscle mass; VFA, visceral fat area; D, HADS-D; SEO, sputum eosinophils; URI, previous upper respiratory infection induced asthma attack.

A nomogram containing the seven variables in the logistic regression model was constructed (Figure 4). The importance of each variable in the full model is illustrated (Figure S2). The SMM level had the third-largest predictive value among the seven variables.

#### 3.5.3. Performance of the Prediction Model and Clinical Applicability of the Nomogram

**Figure 5:**
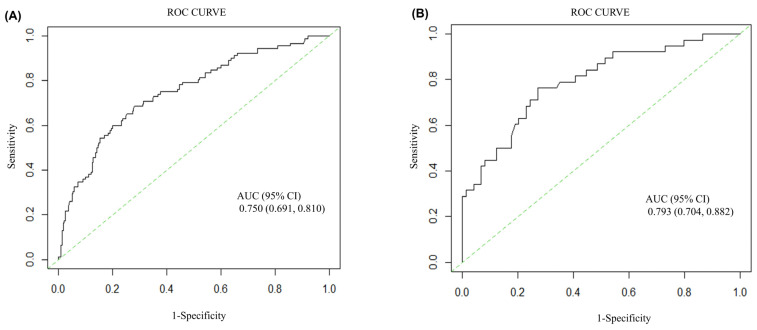
Receiver operating characteristic (ROC) curve for the prediction model for AEx in the training (**A**) and validation (**B**) cohorts. The x-axis, labeled specificity, represents the true-negative rate. The y-axis, labeled sensitivity, represents the true-positive rate. The area under the curve (AUC) and the 95% confidence interval (CI) are shown in the graph. AEx, asthma exacerbation.

There were no significant differences in sociodemographic characteristics between the training and validation cohorts (Tables S7 and S8). The C-indices of the nomogram in the training and validation cohorts were 0.750 (95% CI: 0.691–0.810) and 0.793 (0.704–0.882), respectively (Figure 5), indicating moderate accuracy. The *p* values of the HL tests (training cohort: *p* = 0.531; validation cohort: *p* = 0.465) indicated a lack of significance, suggesting no evidence of poor goodness-of-fit for the prediction model in the two cohorts. Likewise, the calibration curves, showed that the observed and predicted future risk of moderate-to-severe AEx in the final multivariate model were in good agreement in the training and validation cohorts (Figure S3). The NRI and IDI values indicated that SMM offered a significant statistical improvement in the performance of the prediction model in both the training and validation cohorts. The NRI was 0.285 (95% CI: 0.061–0.508; *p* = 0.013) in the training cohort (Table S9), whereas in the validation cohort, the NRI was 0.481 (0.100–0.861, *p* = 0.013). Similar results were obtained using IDI.

Finally, the decision curve showed that using the AEx nomogram model (C) to predict AEx added more benefits than either model (A) or model (B) with different threshold probabilities (Figure S4A). In addition, with a threshold of 0.1 to 0.65, model (C) had the maximum benefits range, as shown in the DCA curve, indicating that SMM played a critical role in the clinical applicability of the prediction model.

The CIC visually shows the proportion of those with true AEx. The CIC also showed that real patients who were at high risk of AEx are included by estimating the number of patients with the risk threshold of 0.1 to 0.65 with the acceptable cost: benefit ratio (Figure S4B).

## 4. Discussion

To the best of our knowledge, this is the first study to investigate the association between SMM and asthma outcome in future. Even though previous studies have explored the relationship between muscle and asthma [47,48], they aimed to evaluate the peripheral muscle strength or the mechanism of reduced SMM in the patients with asthma, and did not observe the association between SMM and asthma-related outcomes in future.

Our study indicated that patients in the SMM ^Low^ group had a lower BMI and more airway obstruction. Moreover, low SMM as an independent risk factor was associated with poor asthma control and an increased risk of AEx. In contrast, SMM ^High^ group patients had less airway obstruction and sputum eosinophils, in association with better asthma control. Additionally, the prediction model involving SMM for predicting AEx established in our study had moderate discrimination, calibration, and clinical utility and demonstrated that SMM played a critical role in the prediction model. Our study indicated that SMM played an important role in asthma control and exacerbation, and reduced SMM could be a potential extra-pulmonary treatable trait to target in clinical asthma management.

In this study, the SMM ^Low^ group had more airway obstruction, demonstrated by reduced pre-bronchodilator FEV_1_% predicted, compared to the SMM ^High^ group. However, there was no difference in pre-bronchodilator FEV_1_/FVC, which is consistent with previously published studies [49]. This could be explained by the fact that FEV_1_ represents the expiration flow rate; therefore, FEV_1_ could be reduced in participants with low SMM because they may have a weakened ability to inflate and deflate their lungs. However, the ratio of FEV_1_/FVC may remain constant regardless of the muscle mass.

Previous study indicated that medication use, ICS in particular, significantly reduced sputum eosinophils [50]. Although no significant difference was found in medication use in the three groups, our study still found that the SMM ^High^ group had a lower sputum eosinophils percentage. Two issues that can be explained as follows: SMM myocytes express and secrete numerous cytokines such as IL-6. Steensberg et al. demonstrated that physiological concentrations of IL-6 induce an anti-inflammatory response in humans [51]. Therefore, although we did not measure inflammatory cytokines, according to previous relevant studies, we speculated that the SMM ^High^ group would have increased levels of anti-inflammatory cytokines in circulation, which could inhibit the recruitment of inflammatory cells in the airway [52]. Conversely, the SMM ^Low^ group patients with higher eosinophilic inflammation needed more ICS, indicating that there may be more severe asthma. Hence, further studies are needed to elucidate the mechanisms by which SMM reduces inflammation in asthma.

Low SMM can be considered a potential extra-pulmonary treatable trait because it is clinically relevant, identifiable, measurable, and treatable [53]. It has been shown that most myokines that are regulated by exercise counteract the detrimental effects of adipokines and have beneficial effects on glucose and lipid metabolism and inflammation [54]. In contrast, physical inactivity and muscle disuse lead to the loss of muscle mass and, consequently, to the activation of a network of inflammatory pathways, which promotes the development of a cluster of chronic diseases. Therefore, SMM could potentially provide an important target for clinicians to guide the non-pharmacological treatment of patients with asthma. For example, increasing physical activity can be used to increase SMM, thereby improving asthma control and reducing exacerbations. This is in agreement with a study by McDonald et al. [18], which identified sarcopenia as a potentially treatable trait of asthma.

As is well known, chronic intake of OCS and ICS induced clinically significant muscle hypotrophy for daily treatment [55]. Furthermore, systemic and oral beta-agonists could increase SMM while there has been no evidence to prove the association between inhaled beta-agonists and SMM [56]. Therefore, ICS/LABA and cumulative doses of OCS equivalent to prednisone, as two important confounders, were adjusted to relevant logistic models. We still found the SMM ^Low^ group was still significantly associated with moderate-to-severe AEx. Accordingly, medication use had no effect on the results. This study has several limitations that need to be addressed. Firstly, BC was measured using BIA only. Dual-energy X-ray absorptiometry is considered a more reliable method for BC assessment. Nevertheless, previous reports have confirmed that multifrequency BIA systems can provide accurate muscle mass and fat mass values that are comparable to those measured using dual-energy X-ray absorptiometry in various populations [57,58]. Secondly, this was a population-based cohort study in China that was specific to the Chinese population. Therefore, the clinical prediction model requires further external validation in multiple centers and different races. Thirdly, although we did not deny that low SMM also could be a consequence of incompletely controlled asthma leading to less physical activity, which formed a vicious circle [2], our prospective cohort study design enabled us to determine the causation that SMM was an exposure factor and AEx were taken as outcomes. Finally, the imbalance of the sample size of a statistical test among the three groups leads to influence the power of a statistical test [59]. Therefore, we calculated the power of the multiple logistic regression for the risk of AEx between the SMM ^Low^ and SMM ^Normal^ groups. However, the power of the multiple logistic regression was 0.8992. Commonly, the value of power is required to be greater than 0.8 [60]. Thus, the imbalance of sample size was not found to have an influence on the power of the risk of AEx between the SMM ^Low^ and SMM ^Normal^ groups in our study.

## 5. Conclusions

We demonstrated that low SMM, as a potential extra-pulmonary treatable trait, is an independent risk factor for asthma control and exacerbation. Furthermore, our model involving SMM for predicting the risk of exacerbation in patients with asthma suggests that SMM may be a suitable therapeutic target for clinical asthma management.

## Figures and Tables

**Table 1:** Demographic and clinical characteristics of the included patients grouped by SMM.

Variables	SMM ^Normal^	SMM ^Low^	SMM ^High^	*χ*^2^/*H*	*p* Value
*n*	223	88	23		
Anthropometric/asthma data					
Age, years, median (Q1, Q3)	44.5 (36.8, 62.0)	57.0 (40.0, 69.0)	47.5 (38.3, 53.0)	0.010	0.995
Female/male, *n* (%)	148 (66.4)/75 (33.6)	58 (65.9)/30 (34.1)	13 (56.5)/10 (43.5)	0.901	0.637
BMI					
kg/m^2^, median (Q1, Q3)	24.09 (22.77, 26.37)	20.66 (19.46, 22.77) *	27.85 (27.29, 30.11) *^,^ **	111.165	<0.001
Normal/overweight/obese, *n* (%)	130 (58.3)/75 (33.6)/17 (7.6)	66 (75.0)/5 (5.7)/0 (0) *	3 (13.0)/8 (34.8)/12 (52.2) *^,^ **	NA	<0.001 §
WHR, median (Q1, Q3)	0.88 (0.84, 0.92)	0.83 (0.79, 0.90) *	0.93 (0.90, 0.95) *^,^ **	29.483	<0.001
Smoking history (*n*), current/ex/never smoker	18/29/176	6/17/65	4/2/17	5.058	0.281
Pack years & median (Q1, Q3)	13.00 (2.50, 26.00)	21.50 (6.63, 32.00)	18.25 (3.50, 32.00)	1.276	0.528
Asthma duration (y), median (Q1, Q3)	5.0 (2.0, 20)	5.0 (2.0, 19.5)	9.0 (2, 25.0)	0.771	0.680
Early-onset asthma #, *n* (%)	38 (17.0)	14 (15.9)	6 (26.1)	NA	0.024 §
Atopy status, *n* (%)	79 (38.3)	35 (44.9)	6 (30.0)	1.812	0.404
Previous upper respiratory infection-induced asthma exacerbations, *n* (%)	155 (69.8)	67 (76.1)	15 (65.2)	1.652	0.438
Asthma family history, *n* (%)	73 (32.7)	37 (42.0)	14 (60.9) *	NA	0.031 §
Spirometry					
Pre-bronchodilator FEV_1_, L, median (Q1, Q3)	2.06 (1.64, 2.85)	1.64 (1.28, 2.49)	2.32 (2.02, 2.65) **	8.632	0.013
Pre-bronchodilator FEV_1_% predicted, median (Q1, Q3)	76.0 (62.0, 90.0)	66.0 (47.0, 82.5) *	92.0 (78.0, 104.0) **	15.684	<0.001
Pre-bronchodilator FEV_1_/FVC, %, median (Q1, Q3)	66.01 (57.54, 76.79)	66.41 (55.23, 75.43)	74.43 (59.28, 82.21)	2.316	0.314
Asthma control					
ACQ-6 scores, median (Q1, Q3)	0.5 (0, 1.3)	0.8 (0.3, 1.3)	0.2 (0, 0.6) **	8.384	0.015
Incompletely controlled asthma, *n* (%) ‡	95 (42.6)	49 (55.7)	5 (21.7) **	9.599	0.008
Health status					
HADS-A					
Median (Q1, Q3)	1.0 (0, 4.0)	1.0 (0, 4.0)	1.0 (0, 2.0)	3.332	0.189
≥8, *n* (%) ¶	15 (6.7)	6 (6.8)	1 (4.3)	NA	1.000 §
HADS-D					0.504
Median (Q1, Q3)	1.0 (0, 3.0)	1.0 (0, 3.0)	0 (0, 2.0)	1.370	0.504
≥8, *n* (%) ¶	16 (7.2)	8 (9.1)	1 (4.3)	NA	0.780 §
Asthma-related medications					
ICS (BDP equivalent) dose (μg/d), median (Q1, Q3)	400.0 (400.0, 1000.0)	500.0 (400.0, 1000.0)	400.0 (200.0, 625.0)	4.064	0.131
ICS/LABA, *n* (%)	135 (60.5)	44 (50.0)	13 (56.5)	2.877	0.237
OCS use					
*n* (%)	8 (3.6)	1 (1.1)	2 (8.7)	NA	0.157 §
Days with OCS use for exacerbation, median (Q1, Q3)	7.00 (6.50, 7.00)	7.00	6.00 (2.00, 10.00)	0.084	0.959
Daily doses of OCS equivalent to prednisone †, mg, median (Q1, Q3)	20.00 (20.00, 32.50)	20.00	55.00 (30.00, 80.00)	3.598	0.165
Cumulative doses of OCS equivalent to prednisone †, mg, median (Q1, Q3)	157.50 (140.00, 280.00)	140.00	430.00 (60.00, 800.00)	0.242	0.886
Leukotriene modifier, *n* (%)	74 (33.2)	24 (27.3)	9 (39.1)	1.584	0.453
Theophylline, *n* (%)	35 (15.7)	19 (21.6)	3 (13.0)	NA	0.428 §
Exacerbation in the past year, *n* (%)					
Severe exacerbation	57 (25.6)	29 (33.0)	10 (43.5)	4.303	0.116
Hospitalization	59 (26.5)	22 (25.0)	3 (13.0)	1.994	0.369
Emergency room visit	31 (13.9)	14 (15.9)	2 (8.7)	NA	0.728 §
Unscheduled visit	68 (30.5)	28 (31.8)	5 (21.7)	0.899	0.638
Comorbidity, *n* (%)					
Rhinitis	129 (57.8)	42 (47.7)	8 (34.8)	6.113	0.047
Bronchiectasis	11 (4.9)	4 (4.5)	0 (0.0)	NA	0.825 §
Sleep apnea	3 (1.3)	0 (0.0)	0 (0.0)	NA	0.646 §
GERD	14 (6.3)	4 (4.5)	1 (4.3)	NA	0.922 §
Eczema	45 (20.2)	17 (19.3)	6 (26.1)	NA	0.750 §
COPD	11 (4.9)	11 (12.5)	0 (0.0)	NA	0.037 §
Diabetes	7 (3.1)	1 (1.1)	1 (4.3)	NA	0.346 §

Abbreviations: SMM, skeletal muscle mass; BMI, body mass index; WHR, waist-to-hip ratio; FEV_1_, forced expiratory volume in 1 s; FVC, forced vital capacity; ACQ, asthma control questionnaire; HADS-A, Hospital Anxiety and Depression Scale-Anxiety; HADS-D, Hospital Anxiety and Depression Scale-Depression; ICS, inhaled corticosteroid; BDP, beclomethasone dipropionate; LABA, long-acting beta-agonist; OCS, oral corticosteroid; GERD, gastroesophageal reflux disease; COPD, chronic obstructive pulmonary disease; NA, not applicable; Q1, first quartile; Q3, third quartile. & Never smokers were excluded from the analysis of pack-years. Pack years: the number of cigarettes smoked per day × years of smoking. ‡ Incompletely controlled asthma: ACQ mean scores ≥ 0.75. ¶ Depression or anxiety disorders was defined as a score ≥ 8 on the respective HADS-D or HADS-A domains. # Early-onset asthma (onset before 12 years of age). † The calculations based on the patients of using OCS in the past year. * *p* < 0.05 vs. SMM ^Normal^, ** *p* < 0.05 vs. SMM ^Low^. The significance level is 0.05. Significance values have been adjusted by the Bonferroni correction for multiple tests. § Fisher’s exact probability.

**Table 2:** Association of SMM with incompletely controlled asthma (ACQ ≥ 0.75) using multivariable logistic regression with adjustment for confounders.

Group	β	SE for β	OR adj	95% CI for OR adj		*p* Value
				Lower	Upper	
SMM ^Normal^			Reference			
SMM ^High^	−0.983	0.523	0.374	0.134	1.044	0.060
SMM ^Low^	0.512	0.255	1.668	1.012	2.749	0.045

Abbreviations: SMM, skeletal muscle mass; ACQ, asthma control questionnaire; OR, odds ratio. Adjusted for age, sex, BMI, ICS/LABA, cumulative doses of OCS equivalent to prednisone, smoking status, severe asthma exacerbation in the past year, forced expiratory volume in 1 s% predicted.

**Table 3:** Asthma exacerbation within the 12-month follow-up period grouped by SMM in the training cohort.

Variables	SMM ^Normal^ Group	SMM ^Low^ Group	SMM ^High^ Group	Total	*χ*^2^/*H*	*p* Value
*n*	213	82	23	318		
Moderate-to-severe exacerbation						
*n* (%)	55 (25.8)	33 (40.2) *	4 (17.4)	92	7.595	0.022
Mean ± SD	0.61 ± 1.40	0.94 ± 1.53 *	0.52 ± 1.24	0.65 ± 1.40	7.149	0.028
Severe exacerbation						
*n* (%)	29 (13.6)	20 (24.4)	0 (0) **	49	NA	0.006 §
Mean ± SD	0.27 ± 0.84	0.43 ± 0.96	0 ± 0 **	0.28 ± 0.83	9.399	0.009

Abbreviations: SMM, skeletal muscle mass; SD, standard deviation; NA, not applicable. * *p* < 0.05 vs. SMM ^Normal^, ** *p* < 0.05 vs. SMM ^Low^. The significance level is 0.05. Significance values have been adjusted by the Bonferroni correction for multiple tests. § Fisher’s exact probability.

## Data Availability

The data used and analyzed during this research are available from the corresponding author upon reasonable request.

## Supplementary Materials

The following supporting information can be downloaded at: https://www.mdpi.com/article/10.3390/jcm11237241/s1, Table S1: Inflammatory characteristics of the included participants in the training cohort grouped by SMM; Table S2: Body composition of the included participants with asthma grouped by SMM; Table S3: Association between SMM and moderate-to-severe asthma exacerbation within the 12-month follow-up period after adjusting for confounders; Table S4: Association between SMM and moderate-to-severe asthma exacerbation within the 12-month follow-up period in sensitive analyses ^#^ (*n* = 252); Table S5: The missing rates of the data set of the variables in our study; Table S6: Correlation between traits, with positive correlations denoted in red and negative correlations in blue (darker = stronger correlation). The symbol (*) placed next to the numbers indicate that the correlation was statistically significant (*p* < 0.05); Table S7: Demographic and clinical characteristics of the included participants in the training and validation cohorts; Table S8: Body composition of the included participants in the training and validation cohorts; Table S9: Assessing improvement of model performance after adding SMM; Figure S1: Predictor selection using the least absolute shrinkage and selection operator (LASSO) binary logistic regression model; Figure S2: The relative importance of variables included in the large model for the prediction of future moderate-to-severe exacerbation of asthma in the following year; Figure S3: Calibration curves for the training (A) and validation (B) cohorts and Figure S4: (A) Decision curves analysis for two risk models for AEx produced with Decision Curve software (B) Clinical impact curve for the SMM-based risk model. Of 1000 patients.

## Abbreviations

AEx	Asthma exacerbation
ACQ	Asthma Control Questionnaire
ACT	Asthma Control Test
ATS	American Thoracic Society
AUC	Receiver operator characteristic (ROC) curve
BC	Body composition
BMI	Body mass index
BIA	Multifrequency bioimpedance analysis
BDP	Beclomethasone dipropionate
COPD	Chronic obstructive pulmonary disease
CIC	Clinical impact curve
C-index	Concordance index
DCA	Decision curve analysis
ER	Emergency room
ERS	European Respiratory Society
FM	Fat mass
FeNO	Fractional exhaled nitric oxide
FEV_1_	Forced expiratory volume in 1 s
FVC	Forced vital capacity
GERD	Gastroesophageal reflux disease
HADS	Hospital Anxiety and Depression Scale
HL	Hosmer–Lemeshow
IgE	Immunoglobulin E
ICS	Inhaled corticosteroid
IDI	Integrated discrimination improvement
LABA	Long-acting β2-agonist
LASSO	Least absolute shrinkage and selection operator
NRI	Net reclassification improvement
OCS	Oral corticosteroid
PBF	Percentage body fat
ROC	Receiver operator characteristic
SMM	Skeletal muscle mass
SPT	Skin prick test
SCS	Systemic corticosteroids
VFA	Visceral fat area
