# Supplementary material for: Reduced Skeletal Muscle Mass Is Associated with an Increased Risk of Asthma Control and Exacerbation
Source: J Clin Med. 2022 Dec 6;11(23):7241. doi: 10.3390/jcm11237241 (PMC9738130; doi:10.3390/jcm11237241)
Supplement: Supplementary file 1 [file jcm-11-07241-s001.zip › jcm-2013584-supplementary.pdf]

Supplementary Materials

This appendix has been included by the authors to provide readers with additional information about the methods and results. The components of the Supplementary Materials are as follows:

Reduced skeletal muscle mass is associated with an increased risk of asthma control and exacerbation

Methods

Data collection

Data on demographics and clinical characteristics were collected using standardized case-report forms. Further assessments included the following.

Sputum induction (SI) and processing

We performed sputum induction and processing based on a standardized operation process as described in our previous studies [1,2]. Sputum was induced using either 4.5% hypertonic saline or saline atomized with an ultrasonic nebulizer (Cumulus, HEYER Medical AG, Rhineland-Palatinate, Germany) in subjects with FEV_1% predicted 40% or greater or FEV_1% predicted less than 40%. Sputum plugs (100 mL) were processed using 400 mL of dithiothreitol and 400 mL of phosphate-buffered saline. Cytospins were prepared using a centrifugation-smear (CytoPro 7620, Wescor, Inc. Logan, UT), stained (May-Grunwald-Giemsa) and differential cell counts were obtained from 400 non-squamous cells. Differential cell counts were performed by well-trained researchers from the University of Newcastle, New South Wales, Australia, and West China Hospital, China.

Depression and anxiety assessment

Depression or anxiety symptoms were assessed using the 14-item Hospital Anxiety and Depression Scale (HADS) and defined as a score of ≥ 8 on the respective HADS-D or HADS-A domains [3].

Blood analysis

Venous blood samples were collected either in ethylenediaminetetraacetic acid-treated tubes for total and differential blood cell counts or in untreated tubes to obtain serum for measurement of total IgE level by immunoassay (Beckman Immage 800 immunoassay analyzer; Beckman Coulter Inc., USA) with a minimum detectable level of IgE of 5.0 IU/mL.

Atopy

As previously described, SPT was performed on the volar surface of the forearm of the subjects by a trained technician [4]. The subjects were pricked with house dust mites (Dermatophagoides pteronyssinus and Dermatophagoides farinae), mold (Alternaria tenuis and Aspergillus species), dog hair, cat hair, pollen (ragweed, birch, and London plane), and cockroaches. Histamine and saline were used as the positive and negative controls, respectively.

Clinical prediction model

Selection of variables and clinical prediction model establishment

Variables with missing data (5%–40%) were imputed using multiple imputation (MI), as described in previous studies [5]. Below 5% of data are negligible and more than 40% of missing data did not use MI.

Nomogram establishment of predicting AEx

A nomogram is a pictorial representation of a complex mathematical formula of multifactor regression analysis, which is widely used as a prognostic device [6]. The total points accumulated by the various covariates corresponded to the predicted probability of AEx for a patient [7]. The advantage of this nomogram is its ability to estimate individualized risk on the basis of the patient and AEx, which can help with clinical decision-making and fulfill personalized medicine.

Performance of the model and clinical applicability of the nomogram

The calibration plot of our prediction model and ROC curves were used to estimate the prediction performance of the nomogram [8].

A nomogram's predictive accuracy (discrimination) was measured using a C-index, which quantifies the level of concordance between predicted probabilities and the actual chance of having an event of interest. The C-index, with its respective CI, provided a more comprehensive measure of discrimination. The area under the ROC curve indicated that a test with an area greater than 0.9 has high accuracy, while 0.7–0.9 indicates moderate accuracy, 0.5–0.7, low accuracy and 0.5 a chance result [8].

The ability of a model to distinguish patients with different outcomes is known as discrimination [9]. The extent to which the predictions are from the actual outcomes is referred to as calibration. The HL goodness-of-fit test and calibration curves were used to assess model calibration [9]. P value > 0.05 for the HL test suggests no evidence of poor goodness-of-fit, which is the desired outcome for a predictive model.

Internal validation of the model was performed using both 1000 bootstrap sampling to produce bias-corrected estimates of the model's performance. Bootstrapping replicates the process of sample generation from an underlying population by drawing samples with replacements from the original dataset of the same size as the original data set. Bootstrapping is considered to be an excellent approach for estimating internal validity because it is a stable and nearly unbiased estimation of model performance [10].

As calibration curve is typically assessed by reviewing the plot of the predicted probabilities from the nomogram versus the actual probabilities. A perfectly accurate nomogram prediction model would result in a plot where the observed and predicted probabilities for the given groups fall along the 45-degree line. The distance between the pairs and the 45-degree line is a measure of the absolute error of the nomogram's prediction [11].

Two ways of assessing the improvement in model performance were accomplished by adding new factors: NRI and IDI. NRI is based on event-specific reclassification tables. IDI is based on the ability of the new model to improve integrated sensitivity without sacrificing integrated specificity.

References for Supplementary Data

1. Liu, L.; Zhang, X.; Liu, Y.; Zhang, L.; Zheng, J.; Wang, J.; Hansbro, P.M.; Wang, L.; Wang, G.; Hsu, A.C. Chitinase-like protein YKL-40 correlates with inflammatory phenotypes, anti-asthma responsiveness and future exacerbations. *Respir. Res.* **2019**, *20*, 95. https://doi.org/10.1186/s12931-019-1051-9.
2. Wang, G.; Baines, K.J.; Fu, J.J.; Wood, L.G.; Simpson, J.L.; McDonald, V.M.; Cowan, D.C.; Taylor, D.R.; Cowan, J.O.; Gibson, P.G. Sputum mast cell subtypes relate to eosinophilia and corticosteroid response in asthma. *Eur. Respir. J.* **2016**, *47*, 1123-1133. https://doi.org/10.1183/13993003.01098-2015.
3. Bjelland, I.; Dahl, A.A.; Haug, T.T.; Neckelmann, D. The validity of the Hospital Anxiety and Depression Scale. An updated literature review. *J. Psychosom. Res.* **2002**, *52*, 69-77. https://doi.org/10.1016/s0022-3999(01)00296-3.
4. Zheng, J.; Zhang, X.; Zhang, L.; Zhang, H.P.; Wang, L.; Wang, G. Interactive effects between obesity and atopy on inflammation: A pilot study for asthma phenotypic overlap. *Ann. Allergy Asthma Immunol.* 2016, 117, 716–717. https://doi.org/10.1016/j.anai.2016.09.430.
5. Jakobsen, J.C.; Gluud, C.; Wetterslev, J.; Winkel, P. When and how should multiple imputation be used for handling missing data in randomised clinical trials - a practical guide with flowcharts. *BMC. Med. Res. Methodol.* **2017**, *17*, 162. https://doi.org/10.1186/s12874-017-0442-1.
6. Pencina, M.J.; D'Agostino, R.B., Sr.; D'Agostino, R.B., Jr.; Vasan, R.S. Evaluating the added predictive ability of a new marker: from area under the ROC curve to reclassification and beyond. *Stat. Med.* **2008**, *27*, 157-172; discussion 207-112. https://doi.org/10.1002/sim.2929.
7. Diblasio, C.J.; Kattan, M.W. Use of nomograms to predict the risk of disease recurrence after definitive local therapy for prostate cancer. *Urology.* **2003**, *62 Suppl 1*, 9-18. https://doi.org/10.1016/j.urology.2003.09.029.
8. Akobeng, A.K. Understanding diagnostic tests 3: Receiver operating characteristic curves. *Acta Paediatr.* 2007, 96, 644–647. https://doi.org/10.1111/j.1651-2227.2006.00178.x.
9. Steyerberg, E.W.; Vergouwe, Y. Towards better clinical prediction models: seven steps for development and an ABCD for validation. *Eur. Heart. J.* **2014**, *35*, 1925-1931. https://doi.org/10.1093/eurheartj/ehu207.
10. Steyerberg, E.W.; Harrell, F.E., Jr.; Borsboom, G.J.; Eijkemans, M.J.; Vergouwe, Y.; Habbema, J.D. Internal validation of predictive models: efficiency of some procedures for logistic regression analysis. *J. Clin. Epidemiol.* **2001**, *54*, 774-781. https://doi.org/10.1016/s0895-4356(01)00341-9.
11. Iasonos, A.; Schrag, D.; Raj, G.V.; Panageas, K.S. How to build and interpret a nomogram for cancer prognosis. *J. Clin. Oncol. Off. J. Am. Soc. Clin. Oncol.* **2008**, *26*, 1364-1370. https://doi.org/10.1200/jco.2007.12.9791.

Supplementary Tables

Table S1. Inflammatory characteristics of the included participants in the training cohort grouped by SMM.

Variables	SMM [Normal]	SMM [Low]	SMM [High]	Total	*H*	*P* value
n	136	55	14	205		
Sputum						
Eosinophils, %, median (Q1, Q3)	0.25 (0, 3.50)	0.25 (0, 0.75)	0 (0, 0.25) *	0.25 (0, 1.75)	7.329	0.029
Neutrophils, %, median (Q1, Q3)	34.00 (15.50, 58.88)	44.75 (17.75, 90.50)	37.25 (22.63, 72.25)	34.50 (14.25, 67.63)	2.810	0.245
Lymphocytes, %, median (Q1, Q3)	0.50 (0.25, 1.13)	0.50 (0.25, 1.38)	0.5 (0, 2.25)	0.50 (0.25, 1.36)	1.172	0.557
Macrophages, %, median (Q1, Q3)	62.25 (31.25, 79.24)	45.50 (7.75, 75.75)	60.75 (35.50, 72.50)	58.75 (22.38, 78.99)	3.332	0.189
FeNO, ppb, median (Q1, Q3)	37.00 (21.00, 67.00)	31.00 (19.50, 72.00)	25.00 (18.50, 45.00)	34.50 (21.00, 67.00)	1.536	0.464
Peripheral blood						
Eosinophils, $\times 10^9$/L, median (Q1, Q3)	0.20 (0.10, 0.41)	0.16 (0.11, 0.31)	0.24 (0.12, 0.47)	0.21 (0.12, 0.40)	2.961	0.227
Neutrophils, $\times 10^9$/L, median (Q1, Q3)	3.36 (2.79, 4.33)	3.56 (2.92, 5.00)	4.03 (3.60, 5.59)	3.53 (2.77 4.50)	3.033	0.219
Lymphocytes, $\times 10^9$/L, median (Q1, Q3)	1.67 (1.41, 1.93)	1.74 (1.43, 2.29)	2.00 (1.66, 2.25)	1.71 (1.41, 2.06)	0.471	0.790
Monocytes, $\times 10^9$/L, median (Q1, Q3)	0.35 (0.27, 0.43)	0.29 (0.35, 0.46)	0.33 (0.29, 0.51)	0.35 (0.28, 0.45)	0.700	0.705
Basophils, $\times 10^9$/L, median (Q1, Q3)	0.04 (0.02, 0.05)	0.03 (0.02, 0.04)	0.03 (0.02, 0.05)	0.04 (0.02, 0.05)	2.474	0.290
IgE, IU/mL, median (Q1, Q3)	108.00 (36.63, 258.70)	75.02 (34.86, 299.00)	105.06 (83.70, 430.35)	103.56 (36.54, 298.50)	3.057	0.217

Abbreviations: SMM, skeletal muscle mass; FeNO, fractional exhaled nitric oxide; Ig, immunoglobulin; NA, not applicable; Q1, first quartile; Q3, third quartile.

*P < 0.05 vs SMM normal; **P < 0.05 vs SMM low. The significance level is 0.05. Significance values have been adjusted by the Bonferroni correction for multiple tests.

Table S2. Body composition of the included participants with asthma grouped by SMM.					
Variables	SMM Normal	SMM Low	SMM High	H/χ^2	*P* value
n	223	88	23		
SMM					
kg	21.80 (20.20, 26.50)	19.25 (17.25, 22.90) *	28.80 (23.45, 31.65) *, **	58.705	<0.001
% total weight	38.81 (35.70, 42.16)	39.26 (35.15, 42.31)	39.51 (35.81, 41.85)	0.826	0.662
Fat					
kg	16.80 (13.65, 20.80)	14.00 (9.75, 17.15) *	19.90 (15.70, 25.60) **	36.918	<0.001
Normal FM, n (%)	80 (35.9)	37 (42.0)	4 (17.4)	4.833	0.089
PBF (%)	28.60 (23.50, 33.80)	25.95 (20.95, 32.50) *	28.80 (20.95, 32.50)	7.046	0.030
Normal PBF, n (%)	59 (26.5)	33 (37.5)	5 (21.7)	4.373	0.112
VFA (cm^2)	73.10 (57.25, 96.85)	62.95 (43.85, 83.85) *	84.50 (59.95, 111.00) **	14.555	0.001
Normal VFA, n (%)	175 (78.5)	75 (85.2)	15 (65.2)	NA	0.092§

Abbreviations: SMM, skeletal muscle mass; FM, fat mass; PBF, percentage body fat; VFA, visceral fat area; NA, not applicable.

*P < 0.05 vs SMM Normal, **P < 0.05 vs SMM Low. The significance level is 0.05. Significance values have been adjusted by the Bonferroni correction for multiple tests.

§ Fisher's exact probability.

Table S3. Association between SMM and moderate-to-severe asthma exacerbation within the 12-month follow-up period after adjusting for confounders.				
Outcomes	Group	Model 1* RR (95%CI)‡	Model 2 † RR_{adj} (95%CI)‡	Model 3 # RR_{adj} (95%CI)‡
Moderate-to-severe exacerbation	SMM ^{Normal}	1 (ref)	1 (ref)	1 (ref)
	SMM ^{High}	0.67 (0.25, 1.52)	0.37 (0.11, 1.11)	0.40 (0.12, 1.13)
	SMM ^{Low}	1.56 (1.09, 2.08)	2.02 (1.35, 2.68)	1.72 (1.19, 2.29)

Abbreviations: SMM, skeletal muscle mass, RR, risk ratio, RR_{adj}, adjusted risk ratio.

*Model was not adjusted for cofounders.

† Model was adjusted for age, sex, BMI, smoking, ICS/LABA, cumulative doses of OCS equivalent to prednisone, pre-bronchodilator FEV_1% and severe asthma exacerbation last year.

# Model was adjusted for age, sex, BMI, smoking, ICS/LABA, cumulative doses of OCS equivalent to prednisone, pre-bronchodilator FEV_1% and severe asthma exacerbation last year, chronic obstructive pulmonary disease, sleep apnea, bronchiectasis, diabetes, obesity, and gastroesophageal reflux disease by backward elimination in a stepwise fashion.

‡RR and 95% confidence intervals (95%CIs) were calculated by logistic regression models.

Table S4. Association between SMM and moderate-to-severe asthma exacerbation within the 12-month follow-up period in sensitive analyses# (*n* = 252).			
Outcomes	Group	Model 1*<br>RR (95%CI)‡	Model 2 †<br>RR_{adj} (95%CI)‡
Moderate-to-severe exacerbation	SMM^{Normal}	1 (ref)	1 (ref)
	SMM^{High}	0.52 (0.13, 1.64)	0.42 (0.09, 1.53)
	SMM^{Low}	1.63 (1.09, 2.26)	1.77 (1.06, 2.53)

Abbreviations: SMM, skeletal muscle mass; RR, risk ratio, RR_{adj}, adjusted risk ratio

Excluding participants with COPD, sleep apnea, bronchiectasis, diabetes, obesity, and gastroesophageal reflux disease (n =66).

*Model was not adjusted for cofounders.

† Model was adjusted for age, sex, BMI, smoking, ICS/LABA, cumulative doses of OCS equivalent to prednisone, pre-bronchodilator FEV_1% and severe asthma exacerbation last year.

‡RR and 95% confidence intervals (95% CIs) were calculated by logistic regression models.

Table S5. The missing rates of the data set of the variables in our study	
Variable	Missing (%)
Sputum eosinophils	40.1%
Sputum neutrophils	40.1%
HADS-A	0.8%
HADS-D	0.8%
IgE	0.6%
Blood eosinophils	0.4%
Sputum neutrophils	0.4%
Pre-bronchodilator FEV_1 % predicted	0.4%
Rhinitis	0.2%
Bronchiectasis	0.2%
Sleep apnea	0.2%
GERD	0.2%
Eczema	0.2%
COPD	0.2%
Diabetes	0.2%
Atopy	0.2%

Table S6. Correlation between traits, with positive correlations denoted in red and negative correlations in blue (darker = stronger correlation). The symbol (*) placed next to the numbers indicate that the correlation was statistically significant ($P < 0.05$).

	Age	Pre-FEV1	Pack-years	HA	BEO	SEO	SNEUT	IgE	Female	AE	Rhi	Naso	GERD	OSA	COPD	Dia	Bro	Atopy	SMM	Fat	VFA	BMI	ACT	FeNO	URI	A	D	Early-onset	
Age		-0.27***	0.2	0.09	-0.19	-0.05	0.16	-0.14	-0.05	0.1	-0.27***	-0.01	0.05	-0.03	0.28***	0.16	0.02	0.21*	-0.02	0.25**	0.17	0.1	-0.01	-0.16	0.11	-0.09	-0.09	-0.22*	0.05
Pre-FEV1	-0.27***		-0.23**	-0.04	-0.01	-0.02	-0.23*	0.05	0.1	-0.05	0.2	-0.02	-0.02	0	-0.24**	-0.06	-0.09	-0.08	0.21*	-0.02	-0.03	0.02	0.13	0.11	-0.19	-0.07	0.02	-0.05	-0.01
Pack-years	0.2***	-0.23***		-0.03	-0.11	-0.05	0.06	-0.06	-0.38***	0.12	-0.21	0.03	-0.03	0.04	0.26***	0.16	-0.05	0.01	-0.01	0.01	0.11	0.15	0.02	-0.05	0.07	0.03	0	-0.13	0.01
HA	0.09	-0.04	-0.03		-0.01	-0.02	-0.03	-0.05	0	0.01	-0.04	-0.03	0.11	-0.01	0.1	-0.02	-0.02	0.05	-0.02	-0.02	-0.03	0.08	0.02	-0.02	-0.01	-0.03	-0.07	-0.04	0.08
BEO	-0.19***	-0.01	-0.11*	-0.01		0.36***	-0.14	0.24**	0.05	0.06	0.23**	0.14	0.06	-0.08	-0.09	-0.07	0.22*	-0.04	0	-0.15	-0.13	-0.09	-0.2	0.31***	-0.13	0.13	0.11	-0.07	-0.01
SEO	-0.05	-0.02	-0.05	-0.02	0.36***		-0.11	-0.01	0.06	0.09	0.06	0	-0.01	-0.06	-0.09	-0.1	0.26***	0.04	0	-0.12	-0.09	-0.01	-0.13	0.22*	0.01	0.08	0.1	-0.03	0.12
SNEUT	0.16**	-0.23***	0.06	-0.03	-0.14**	-0.11*		-0.06	-0.1	-0.01	-0.15	0	-0.02	0.02	0.08	0.09	-0.11	0.12	-0.08	0.05	0.07	0.03	-0.07	-0.07	-0.02	-0.1	-0.14	-0.04	0.01
IgE	-0.14*	0.05	-0.06	-0.05	0.24***	-0.01	-0.06		-0.03	-0.1	0.09	0.04	-0.07	-0.03	-0.03	0.04	-0.03	-0.3***	-0.05	-0.13	-0.14	0.01	0.05	0.13	-0.09	-0.02	-0.03	-0.01	-0.03
Female	-0.05	0.1	-0.38***	0	0.05	0.06	-0.1	-0.03		-0.05	0.02	-0.1	-0.01	0	-0.14	-0.07	0.04	0.01	-0.03	-0.02	0.11	-0.07	-0.02	-0.12	-0.04	0	0	0.1	0.07
AE	0.1	-0.05	0.12*	0.01	0.06	0.09	-0.01	-0.1	-0.05		0.03	0	0.04	-0.06	0.12	-0.02	0.12	0.11	0	0.02	0.05	0.04	-0.09	0.02	0.13	0.06	0.06	-0.06	0.26***
Rhi	-0.27***	0.2***	-0.21***	-0.04	0.23***	0.06	-0.15**	0.09	0.02	0.03		0.22*	0.07	0.09	-0.04	-0.03	0.06	-0.19	0.01	-0.04	-0.08	-0.01	0	0.2	-0.06	0.11	0.15	0.03	0.09
Naso	-0.01	-0.02	0.03	-0.03	0.14**	0	0	0.04	-0.1	0	0.22***		0.02	0.09	0.09	0.02	0.2	0	0.03	-0.02	-0.06	-0.02	0.07	0.04	0.05	0.11	0.1	-0.02	0.08
GERD	0.05	-0.02	-0.03	0.11*	0.06	-0.01	-0.02	-0.07	-0.01	0.04	0.07	0.02		0.11	0.14	0.04	0.13	0.07	0.02	0.06	0.06	-0.03	-0.03	-0.06	-0.02	0.05	0.03	0.02	0.02
OSA	-0.03	0	0.04	-0.01	-0.08	-0.06	0.02	-0.03	0	-0.06	0.09	0.09	0.11*		-0.03	-0.02	0.13	-0.01	0.03	-0.07	-0.03	-0.03	0.07	0.06	-0.08	-0.07	-0.06	0.04	-0.06
COPD	0.28***	-0.24***	0.26***	0.1	-0.09	-0.09	0.08	-0.03	-0.14*	0.12*	-0.04	0.09	0.14**	-0.03		0.1	0.18	-0.02	-0.15	0.03	0.05	0	-0.05	-0.1	0.12	-0.03	-0.04	-0.09	0.08
Dia	0.16**	-0.06	0.16**	-0.02	-0.07	-0.1	0.09	0.04	-0.07	-0.02	-0.03	0.02	0.04	-0.02	0.1		-0.04	-0.06	0.06	0.02	0.06	0.08	0.13	-0.09	-0.06	-0.08	-0.04	0.02	0.02
Bro	0.02	-0.09	-0.05	-0.02	0.22***	0.26***	-0.11	-0.03	0.04	0.12*	0.06	0.2***	0.13*	0.13*	0.18**	-0.04		0.12	-0.03	-0.09	-0.07	-0.07	-0.03	0	0.11	0.16	0.12	-0.06	0.06
Atopy	0.21***	-0.08	0.01	0.05	-0.04	0.04	0.12*	-0.3***	0.01	0.11*	-0.19***	0	0.07	-0.01	-0.02	-0.06	0.12*		-0.11	0.02	0.09	-0.01	-0.03	-0.05	-0.06	-0.01	0.03	-0.13	0.01
SMM	-0.02	0.21***	-0.01	-0.02	0	0	-0.08	-0.05	-0.03	0	0.01	0.03	0.02	0.03	-0.15**	0.06	-0.03	-0.11*		0.29***	0.16	0.35***	0.08	-0.03	-0.07	-0.08	-0.05	0.05	-0.14
Fat	0.25***	-0.02	0.01	-0.02	-0.15**	-0.12*	0.05	-0.13*	-0.02	0.02	-0.04	-0.02	0.06	-0.07	0.03	0.02	-0.09	0.02	0.29***		0.66***	0.22*	0.01	-0.13	0.12	-0.05	-0.06	0.1	0.03
VFA	0.17**	-0.03	0.11*	-0.03	-0.13*	-0.09	0.07	-0.14**	0.11	0.05	-0.08	-0.06	0.06	-0.03	0.05	0.06	-0.07	0.09	0.16**	0.66***		0.27***	-0.02	-0.15	0.11	0.08	0.02	0.08	0.11
BMI	0.1	0.02	0.15**	0.08	-0.09	-0.01	0.03	0.01	-0.07	0.04	-0.01	-0.02	-0.03	-0.03	0	0.08	-0.07	-0.01	0.35***	0.22***	0.27***		0.05	-0.06	0.03	-0.02	0.02	-0.06	0.02
ACT	-0.01	0.13*	0.02	0.02	-0.2***	-0.13*	-0.07	0.05	-0.02	-0.09	0	0.07	-0.03	0.07	-0.05	0.13*	-0.03	-0.03	0.08	0.01	-0.02	0.05		-0.18	-0.03	-0.2	-0.16	-0.02	-0.09
FeNO	-0.16**	0.11*	-0.05	-0.02	0.31***	0.22***	-0.07	0.13*	-0.12*	0.02	0.2***	0.04	-0.06	0.06	-0.1	-0.09	0	-0.05	-0.03	-0.13*	-0.15**	-0.06	-0.18**		0.01	-0.01	-0.04	-0.04	0.04
URI	0.11*	-0.19***	0.07	-0.01	-0.13*	0.01	-0.02	-0.09	-0.04	0.13*	-0.06	0.05	-0.02	-0.08	0.12*	-0.06	0.11	-0.06	-0.07	0.12*	0.11	0.03	-0.03	0.01		0.05	0	0.08	0.23**
A	-0.09	-0.07	0.03	-0.03	0.13*	0.08	-0.1	-0.02	0	0.06	0.11*	0.11	0.05	-0.07	-0.03	-0.08	0.16**	-0.01	-0.08	-0.05	0.08	-0.02	-0.2***	-0.01	0.05		0.7***	0	0.07
D	-0.09	0.02	0	-0.07	0.11*	0.1	-0.14*	-0.03	0	0.06	0.15**	0.1	0.03	-0.06	-0.04	-0.04	0.12*	0.03	-0.05	-0.06	0.02	0.02	-0.16**	-0.04	0	0.7***		-0.01	0.12
Early-onset	-0.22***	-0.05	-0.13*	-0.04	-0.07	-0.03	-0.04	-0.01	0.1	-0.06	0.03	-0.02	0.02	0.04	-0.09	0.02	-0.06	-0.13*	0.05	0.1	0.08	-0.06	-0.02	-0.04	0.08	0	-0.01		-0.02
	0.05	-0.01	0.01	0.08	-0.01	0.12*	0.01	-0.03	0.07	0.26***	0.09	0.08	0.02	-0.06	0.08	0.02	0.06	0.01	-0.14*	0.03	0.11	0.02	-0.09	0.04	0.23***	0.07	0.12*	-0.02	

HA: heart attack; BEO, blood eosinophils; SEO, sputum eosinophils; SNEUT, sputum neutrophilic; AE, asthma severe exacerbation in the past year; Rhi, rhinitis; Naso, nasosinusitis; GERD, gastroesophageal reflux disease; OSA, obstructive sleep apnea; Dia, diabetes; Bro, bronchiectasis; URI, upper respiratory infection-induced asthma attack; Fat, fat mass; SMM, skeletal muscle mass; VFA, visceral fat area; BMI, body mass index; ACT, Asthma Control Test; FeNO, fractional exhaled nitric oxide; HADS-A, Hospital Anxiety and Depression scale-anxiety; HADS-D, Hospital Anxiety and Depression scale-depression; Early-onset, early-onset asthma.

Table S7. Demographic and clinical characteristics of the included participants in the training and validation cohorts.				
Variables	Training cohort	Validation cohort	χ^2/U	*P* value
	334	157	28524.000	0.116
Anthropometric /asthma data				
Age, years, median (Q1, Q3)	44.0 (35.0, 55.0)	47.0 (36.0, 55.5)	0.022	0.484
Female, n (%)	219 (65.6)	104 (66.2)	10.322	0.006
BMI, kg/m^2, median (Q1, Q3)	23.02 (20.99, 25.02)	23.15 (21.90, 25.00)	28131.500	0.192
WHR, median (Q1, Q3)	0.87 (0.82, 0.92)	0.89 (0.83, 0.94)	26105.500	0.075
Smoking history (n), current/ex/never smoker	28/48/258	0/9/103	14.268	<0.001
Pack-years, median (Q1, Q3) †	14.50 (3.13, 29.00)	15.50 (5.90, 31.25)	1344.500	0.734§
Asthma duration (y), median (Q1, Q3)	5.0 (2.0, 20.0)	10.0 (3.0, 23.0)	16233.5	0.036
Early-onset asthma, n (%)	58 (17.4)	31 (19.7)	0.449	0.530
Atopic status, n (%)	120 (35.9)	147 (43.9)	3.071	0.082
Upper respiratory infection induced asthma attack, n (%)	237 (71.0)	110 (70.1)	0.054	0.832
Asthma family history, n (%)	124 (38.5)	45 (28.7)	4.482	0.034
Spirometry, median (Q1, Q3) (n = 332)				
Pre-bronchodilator-FEV$_1$, L	2.08 (1.56, 2.68)	2.12 (1.57, 2.75)	26561.000	0.732
Pre-bronchodilator-FEV$_1$, % predicted	74.0 (59.0, 88.0)	76.0 (63.0, 89.5)	27724.000	0.255
Pre-bronchodilator-FEV$_1$/FVC, %	67.71 (57.83, 76.76)	68.50 (61.35, 76.28)	27553.500	0.307
Health Status				
HADS-A, median (Q1, Q3)	1.0 (0, 4.0)	1.0 (0, 3.5)	23912.000	0.176

HADS-D, median (Q1, Q3)	1.0 (0, 3.0)	1.0 (0, 3.0)	23816.500	0.219
Asthma-related medication				
ICS (BDP equivalent) dose (μg/d), median (Q1, Q3)	400.0 (400.0, 1000.0)	400.0 (400.0, 700.0)	7208.000	0.396
ICS/LABA, n (%)	192 (57.7)	96 (61.1)	0.721	0.396
OCS, n (%)	11 (3.3)	3 (1.9)	0.299	0.585
Leukotriene modifier, n (%)	107 (32.0)	66 (42.0)	4.912	0.027
Theophylline, n (%)	57 (17.1)	14 (8.9)	5.619	0.019
Comorbidities, n (%)				
Rhinitis	179 (53.6)	103 (66.2)	7.449	0.008
Nasal polyps	28 (8.4)	18 (11.5)	1.244	0.265
Bronchiectasis	15 (4.5)	6 (3.8)	0.108	0.816
Sleep apnea	3 (0.9)	2 (1.3)	0.000	1.000
GERD	19 (5.7)	9 (5.7)	0.001	1.000
COPD	22 (6.6)	7 (4.5)	0.842	0.359
Eczema	68 (20.4)	17 (10.8)	0.000	1.000
Diabetes	9 (2.7)	5 (3.2)	0.001	0.980
Exacerbations in the past year				
Severe exacerbation	96 (28.7)	41 (26.1)	0.367	0.590
Hospitalization	84 (25.1)	30 (19.1)	2.087	0.169
Emergency room visit	47 (14.1)	19 (12.1)	0.365	0.551
Unscheduled visit	101 (30.2)	50 (31.8)	0.164	0.753
Comorbidities, n (%)				
Rhinitis	179 (53.6)	103 (66.2)	7.449	0.008
Nasal polyps	28 (8.4)	18 (11.5)	1.244	0.265

Bronchiectasis	15 (4.5)	6 (3.8)	0.108	0.816
Sleep apnea	3 (0.9)	2 (1.3)	0.000	1.000
GERD	19 (5.7)	9 (5.7)	0.001	1.000
COPD	22 (6.6)	7 (4.5)	0.842	0.359
Eczema	68 (20.4)	17 (10.8)	0.000	1.000
Diabetes	9 (2.7)	5 (3.2)	0.001	0.980

BDP, Beclomethasone dipropionate; BMI, body mass index; WHR, waist-to-hip ratio; FeNO, fractional exhaled nitric oxide; FEV_1, forced expiratory volume in 1 s; FVC, forced vital capacity; FM, fat mass; GERD, gastroesophageal reflux disease; HADS-A, Hospital Anxiety and Depression scale-anxiety; HADS-D, Hospital Anxiety and Depression scale-depression; ICS, inhaled corticosteroid; LABA, long-acting beta-agonist; OCS, oral corticosteroid.

Q1, first quartile; Q3, third quartile.

† Never smokers were excluded from the analysis of pack-years. Pack years: the number of cigarettes smoked per day × years of smoking.

Table S8. Body composition of the included participants in the training and validation cohorts.				
Variables, median (Q1, Q3)	Training cohort (n=334)	Validation cohort (n=157)	U/χ^2	*P* value
SMM (kg)				
SMM, (kg)	21.70 (19.50, 26.10)	21.40 (19.85, 26.35)	26349.000	0.929
Normal SMM, n (%)	223 (66.8)	126 (80.3)	9.452	0.003
SMM (% total weight)	38.96 (35.61, 42.20)	38.50 (35.53, 41.69)	24945.000	0.385
Fat				
Fat mass (kg)	16.30 (12.90, 20.03)	17.50 (13.70, 21.05)	29136.000	0.047
Normal fat mass, n (%)	121 (36.2)	52 (33.1)	0.452	0.544
PBF (%)	27.85 (22.90, 33.63)	29.10 (24.50, 34.35)	28221.000	0.172
Normal PBF, n (%)	95 (28.6)	38 (24.2)	1.047	0.329
VFA, cm^2	70.75 (54.75, 96.23)	76.00 (53.65, 97.45)	27555.000	0.362
Normal VFA, n (%)	265 (79.3)	122 (77.7)	0.171	0.723

FM, fat mass; SMM, skeletal muscle mass; VFA, visceral fat area; PBF, percentage body fat.

Q1, first quartile; Q3, third quartile.

Table S9. Assessing improvement of model performance after adding SMM.				
	Training cohort		Validation cohort	
Prediction model	NRI (95% CI)	*P* value	NRI (95% CI)	*P* value
Model 1	Reference model	NA	Reference model	NA
Model 2	0.285 (0.061–0.508)	0.013	0.481 (0.100–0.861)	0.013

Model 1 represents all risk factors the AE nomogram with subtraction of SMM; Model 2 contains all risk factors of the AE nomogram. Risk factors of AE nomogram contain asthma exacerbation in the past year or not, previous upper respiratory infection induced asthma attack, sputum eosinophils (%), rhinitis, HADS-D (scores), VFA (cm^2) and SMM (kg).

NRI, continuous net reclassification improvement index; 95% CI, 95% confidence interval; NA, not applicable.

Supplementary Figure.

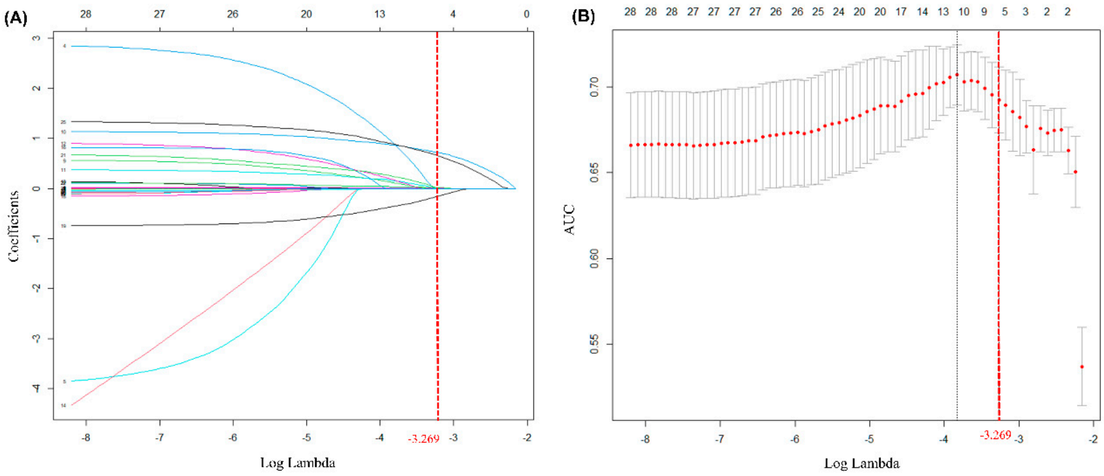

Figure S1. Predictor selection using the least absolute shrinkage and selection operator (LASSO) binary logistic regression model. (A) The tuning of the parameter (λ) for the LASSO model through the 5-fold cross-validation was plotted as a function of log(λ). The y-axis represents partial likelihood deviance, and the lower x-axis represents log(λ). Numbers along the upper x-axis represent the average number of predictors. Red dots indicate the average deviance values for each model with a given λ, where the model provides its best fit of the data. Dotted vertical lines coincide with two special values along the λ sequence. The black dotted vertical line coincides with the value of λ that gives a minimum mean cross validated error. The blue dotted vertical line coincides with the value of λ that gives the most regularized model such that the cross-validated error is within one standard error of the minimum. A λ value of 0.0380, with log (λ) of -3.269, was chosen according to the 5-fold cross-validation. (B) LASSO coefficient profiles of the 28 baseline features. Each curve corresponds to a variable. A coefficient profile plot was generated against the log (λ) sequence. The black dotted vertical line coincides with the value selected using tenfold cross-validation in Figure 3A, where the optimal λ (0.0380, with log (λ) of -3.269) resulted in 7 nonzero coefficients. LASSO, least absolute shrinkage and selection operator.

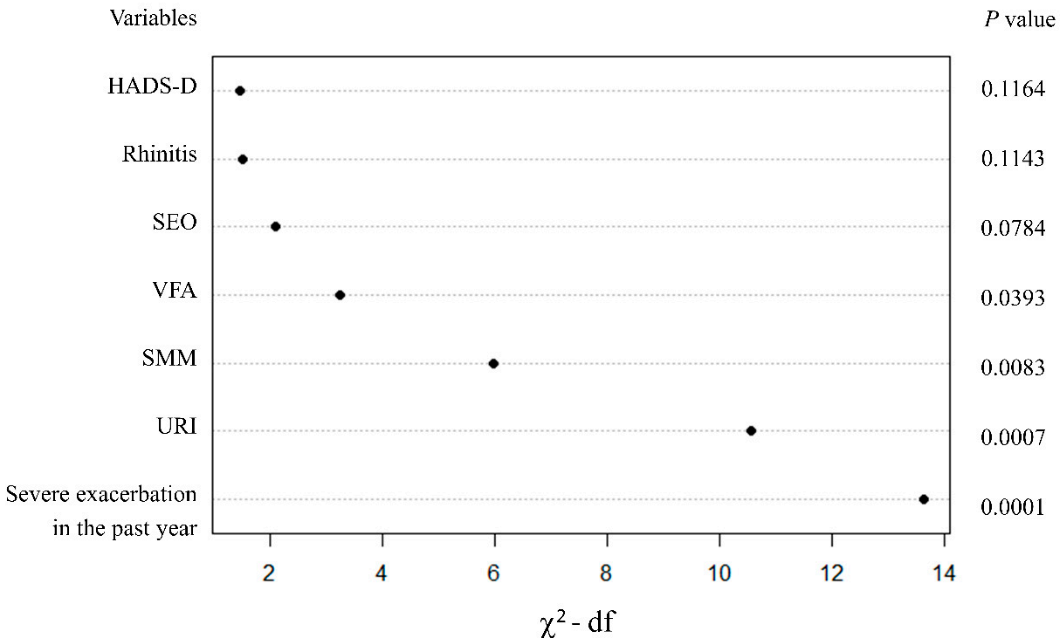

Figure S2. The relative importance of variables included in the large model for the prediction of future moderate-to-severe exacerbation of asthma in the following year is shown, where importance is measured as a chi-square statistic minus the predictor degrees of freedom (df). SMM, skeletal muscle mass; VFA, visceral fat area; D, HADS-D; SEO, sputum eosinophils; URI, previous upper respiratory infection induced asthma attack.

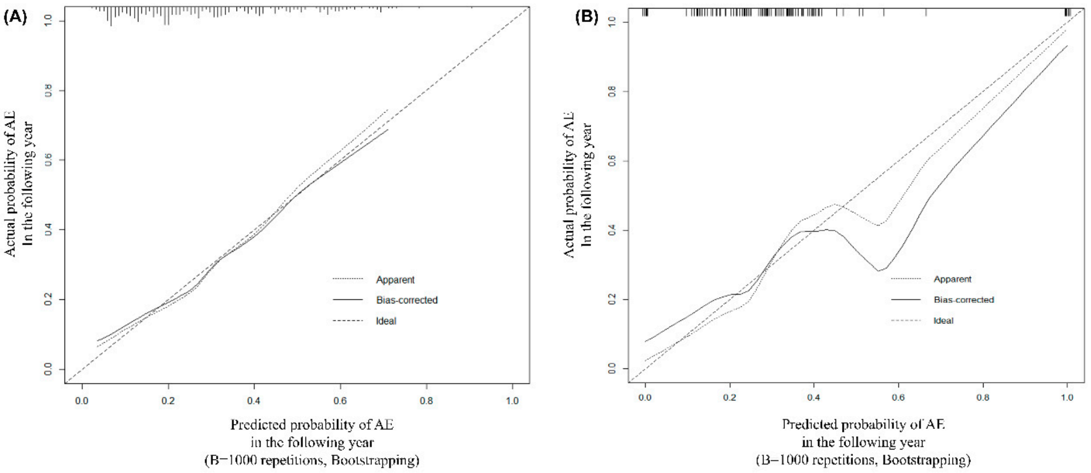

Figure S3. Calibration curves for the training (A) and validation (B) cohorts. The x-axis represents the predicted probability of AEx in the following year. The y-axis represents the actual probability of AEx in the following year. The diagonal dotted line (Ideal) represents a perfect prediction by an ideal model. Apparent is the uncalibrated prediction curve illustrating the accuracy of the original prediction model. The adjusted bias-corrected line (based on the bootstrapping) represents the performance of the model. A closer fit of this adjusted bias-correct line to the diagonal dotted line represents a better prediction. AEx, asthma exacerbation.

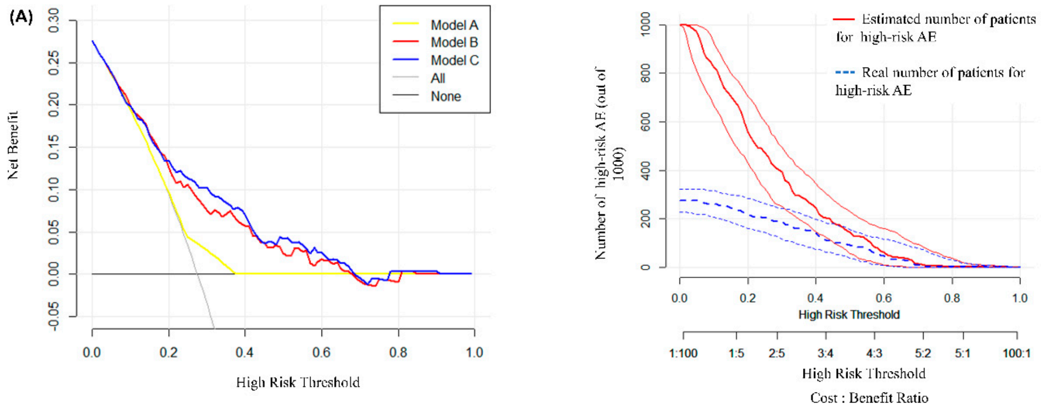

Figure S4. (A) Decision curves analysis for two risk models for AEx produced with Decision Curve software. The vertical axis displays standardized net benefit. The two horizontal axes show the correspondence between risk threshold and cost: benefit ratio. Model A represents only SMM. The blue line represents this AEx nomogram with subtraction of SMM. Model C represents this AEx nomogram. (B) Clinical impact curve for the SMM-based risk model. Of 1,000 patients, the red solid line shows the total number who would be deemed high risk for each risk threshold. The blue dashed line shows how many of those would be true positives (cases). Bands on plots represent pointwise 95% CIs constructed via bootstrapping.
